# Sex differences in COVID-19 mortality in the Netherlands

**DOI:** 10.1007/s15010-021-01744-0

**Published:** 2022-02-09

**Authors:** Annabel Niessen, Anne C. Teirlinck, Scott A. McDonald, Wim van der Hoek, Rianne van Gageldonk-Lafeber, RIVM COVID-19 epidemiology, surveillance group, Mirjam J. Knol

**Affiliations:** Rijksinstituut Voor Volksgezondheid en Milieu (National Institute for Public Health and Environment, RIVM), Bilthoven, The Netherlands

**Keywords:** SARS-CoV-2, Sex-differences, COVID-19

## Abstract

**Introduction:**

Since the first reports of COVID-19 cases, sex-discrepancies have been reported in COVID-19 mortality. We provide a detailed description of these sex differences in relation to age and comorbidities among notified cases as well as in relation to age and sex-specific mortality in the general Dutch population.

**Methods:**

Data on COVID-19 cases and mortality until May 31st 2020 was extracted from the national surveillance database with exclusion of healthcare workers. Association between sex and case fatality was analyzed with multivariable logistic regression. Subsequently, male–female ratio in standardized mortality ratios and population mortality rates relative to all-cause and infectious disease-specific mortality were computed stratified by age.

**Results:**

Male–female odds ratio for case fatality was 1.33 [95% CI 1.26–1.41] and among hospitalized cases 1.27 [95% CI 1.16–1.40]. This remained significant after adjustment for age and comorbidities. The male–female ratio of the standardized mortality ratio was 1.70 [95%CI 1.62–1.78]. The *population* mortality rate for COVID-19 was 35.1 per 100.000, with a male–female rate ratio of 1.25 (95% CI 1.18–1.31) which was higher than in all-cause population mortality and infectious disease mortality.

**Conclusion:**

Our study confirms male sex is a predisposing factor for severe outcomes of COVID-19, independent of age and comorbidities. In addition to general male–female-differences, COVID-19 specific mechanisms likely contribute to this mortality discrepancy.

**Supplementary Information:**

The online version contains supplementary material available at 10.1007/s15010-021-01744-0.

## Introduction

Since the start of the pandemic in December 2019, COVID-19 has spread to most countries in the world, resulting in over 83 million cases and more than 1.8 million deaths by January 2021 [1]**.** The most important risk factor for dying from COVID-19 seems to be older age [2]. Furthermore, certain comorbidities, mainly obesity, cardiovascular disease, hypertension and diabetes, have been associated with severe disease outcome [2–7]**.** Already since the first reports of COVID-19 cases in China, a discrepancy has been reported between males and females in COVID-19 severity and mortality to the detriment of male sex [8, 9]. This difference between males and females has also been described previously in the SARS and MERS epidemics [10, 11]. An analysis of sex-disaggregated data of COVID-19 mortality in 84 countries shows higher case fatality ratios (CFR) in men in the vast majority of the countries, with the Netherlands having one of the highest male:female ratio of CFRs during the first wave of the COVID-19 pandemic [12].

A number of hypotheses have been proposed regarding the underlying mechanisms of these sex differences in COVID-19 mortality. However, male–female disparities in mortality in general are already a known phenomenon. The male–female health-survival paradox describes this phenomenon of a higher life expectancy in females, compared to males, at the expense of higher morbidity, especially at older ages [13]. Although differences in COVID-19 mortality between males and females are striking and have been reported several times [14–17], only a few reports put this in perspective of general population mortality [18, 19]. Moreover, little data have been published on the differences in comorbidities and age distribution of male and female COVID-19 patients and the extent to which these factors influence the risk of dying. Here, we provide a detailed description of the male–female differences in COVID-19 mortality during the first wave of the COVID-19 pandemic in the Netherlands, to gain further insight into the contributing factors. Therefore, we describe male–female differences in COVID-19 mortality in relation to age and comorbidities in the notified cases as well as in relation to age- and sex-specific mortality in the general Dutch population.

## Methods

### Data collection

Data on COVID-19 cases were extracted from the national infectious disease mandatory surveillance database. On January 28, COVID-19 was classified as a mandatory notifiable disease and since then data on patient demographics, comorbidities, source and contact monitoring, hospitalization and possible fatal outcome had to be reported by regional public health services for every laboratory confirmed COVID-19 case. Nursing home residency was estimated based on age and postal code. A confirmed COVID-19 case was defined as a person with a positive PCR result for SARS-CoV-2. The first case in the Netherlands was reported on February 27th. The initial instructions by the Dutch National Institute for Public Health and the Environment (RIVM) was to test hospitalized patients with COVID-19-like symptoms and symptomatic people with a travel history in risk areas or contact history with a laboratory confirmed patient. Early on, this was extended to pneumonia patients in whom no causative agent could be detected and who did not respond to treatment. Due to the rapidly increasing incidence of cases and the scarcity of diagnostic tests, from March 13th testing policy was restricted to suspected cases above 70 years of age and persons with a probable chronical illness, health care workers and patients admitted to the hospital [20]. In clusters of cases, such as in nursing homes, only the first cases were confirmed by PCR. Over time, test capacity grew and testing criteria were broadened and since June 1st, all Dutch citizens can be tested for SARS-CoV-2 in drive-through testing facilities if they have COVID-19-like symptoms.

### Study population

All SARS-CoV-2 positive cases registered in the national infectious diseases electronic notification database until May 31st 2020 were included for analysis. After this date, as a result of the change in testing policy, data were not comparable to the period before May 31st. Data were last updated on August 22th 2020, to include additions or corrections that could have been made to these notifications afterwards. Healthcare workers were excluded from the analysis since they were over-represented in the data due to the testing policy and are not representative of the sex distribution in the general population. Also, notifications with missing data on sex or age were excluded. Due to the large amount of COVID-19 cases, registration requirements were diminished and data on comorbidities were no longer available for all patients after April 10th 2020. Analyses are performed for the study population in total, for the subgroup of hospitalized patients and for a subset of cases registered before April 10th with complete data on comorbidities.

### Outcome definition

Whether a notified case had died was reported by the regional public health services. However, the time that cases were followed-up after initial notification, to check for disease outcome, might have differed between regions. Some, but not all regions updated their notifications with information on deaths in the vital registries of municipalities.

### Analysis

Differences between males and females for categorical baseline variables were analyzed using the X^2^-test. Age was expressed in median (IQR) and analyzed with the Mann–Whitney *U*-test. Mortality within all notified cases (case fatality ratio, CFR) and mortality within hospitalized notified cases was stratified by 5 year age-groups and sex. The association between sex and mortality was assessed with logistic regression, expressed as an odds ratio (OR) and adjusted for age and nursing home residency as a proxy for frailty. For the subset of cases registered before April 10th, comorbidities were added to the model. The model was also fitted to both sexes separately to indicate differences in the contribution of comorbidities to mortality. To account for anticipated sex-related mortality rate differences in the general population, standardized mortality ratios (SMR) were calculated using indirect standardization by dividing the number of deaths in notified cases by the expected number of deaths in notified cases based on population mortality in 2019. SMRs were adjusted for age and reported as a male–female ratio by 5 year age group. 95% confidence intervals were derived through bootstrapping with 10,000 iterations.

To account for male–female population size differences, we additionally described the number of deaths per 100,000 inhabitants (population mortality rate), reflecting the per-capita risk of becoming infected and dying from infection. Population mortality rates were stratified by 5-year age group and sex. Additionally, male–female mortality rate ratio was calculated for nursing home residents. Male–female mortality rate ratios were compared with rate ratios of all-cause mortality, infectious diseases-specific mortality (ICD10: A00-B99), mortality by pneumonia (ICD10: J12-J18) and mortality by influenza (J09-J11) in the general population over the past 5 years (2015 to 2019). Population numbers for 2020 and population mortality numbers for 2019, stratified by age in 5-year intervals, were obtained from Statistics Netherlands [21]. In addition, the distribution of the absolute mortality over time was analyzed and depicted as the moving average over 14 days, stratified by age and nursing home residency. Data analysis was performed in R (version 4.0.0).

## Results

### Characteristics of notified cases

By May 31st 2020 46,507 cases of COVID-19 were registered, of which 16,906 cases concerned health care workers and were excluded from analyses. Furthermore, 62 cases with missing data on age or sex were excluded (0.2%), (supplement A). The remaining study population consisted of 29,539 cases of which 49.5% were males. Of these cases, 11,227 were hospitalized of which 62.9% were males. An overview of the main characteristics of the study population is presented in Table 1. The median age of notified COVID-19 cases was 78 [IQR, 60–87] in females and 71 [IQR, 56–81] in males. Most pronounced was the higher proportion of notified cases aged over 85 in females compared to males. Age-difference between males and females was less pronounced in the hospitalized subgroup. The proportion living in an institution (e.g. nursing home) was much larger in females than in males (37.4 versus 18.8%). Most frequently reported comorbidities were cardiovascular disease or hypertension (39.5%), chronic lung disease (18.6%) and diabetes (17.2%). There were no consistent differences between males and females in the presence of comorbidities.

**Table 1:** Patient characteristics of all notified COVID-19 cases and of hospitalized cases, excluding health care workers, the Netherlands, 28 February–1 June 2020, *n* = 29539

	All cases (*N* = 29,539)			Hospitalised cases (*N* = 11,227)		
	Male	Female	*p*	Male	Female	*p*
*n*	14,627	14,912		7057	4170	
Age (median [IQR])	71.00 [56.00, 81.00]	78.00 [60.00, 87.00]	< 0.001	69.00 [58.00, 77.00]	71.00 [59.00, 79.00]	< 0.001
Age (%)						
0–54	3213 (22.0)	2920 (19.6)	< 0.001	1271 (18.0)	793 (19.0)	< 0.001
55–69	3814 (26.1)	2432 (16.3)		2359 (33.4)	1167 (28.0)	
70–84	5274 (36.1)	4770 (32.0)		2838 (40.2)	1702 (40.8)	
85 +	2326 (15.9)	4790 (32.1)		589 (8.3)	508 (12.2)	
Nursing home resident	2745 (18.8)	5582 (37.4)	< 0.001	214 (3.0)	254 (6.1)	< 0.001
Hospital admission	7057 (48.2)	4170 (28.0)	< 0.001	–	–	–
Comorbidity data available (*n*)*	7395	5949		4811	2716	
Any comorbidity	5530 (74.8)	4696 (78.9)	< 0.001	3565 (74.1)	2149 (79.4)	< 0.001
Cardiovascular disease or hypertension	2958 (40.0)	2308 (38.8)	0.163	2013 (41.8)	1026 (37.2)	< 0.001
Diabetes	1293 (17.5)	1006 (16.9)	0.395	890 (18.5)	504 (18.3)	0.843
Liver disease	63 (0.9)	43 (0.7)	0.461	49 (1.0)	18 (0.7)	0.132
Chronic neuromuscular disease	606 (8.2)	617 (10.4)	< 0.001	234 (4.9)	144 (5.2)	0.523
Immunodeficiency	90 (1.2)	61 (1.0)	0.338	61 (1.3)	33 (1.2)	0.874
Renal impairment	478 (6.5)	428 (7.2)	0.102	293 (6.1)	203 (7.4)	0.035
Chronic lung disease	1335 (18.1)	1145 (19.2)	0.082	914 (19.0)	635 (23.0)	< 0.001
Malignancy	628 (8.5)	486 (8.2)	0.523	405 (8.4)	252 (9.1)	0.300
Other underlying disease	1099 (14.9)	1103 (18.5)	< 0.001	677 (14.1)	545 (19.8)	< 0.001

^*^In some cases more than one comorbidity has been reported

### Mortality among notified COVID-19 cases

**Fig. 1:**
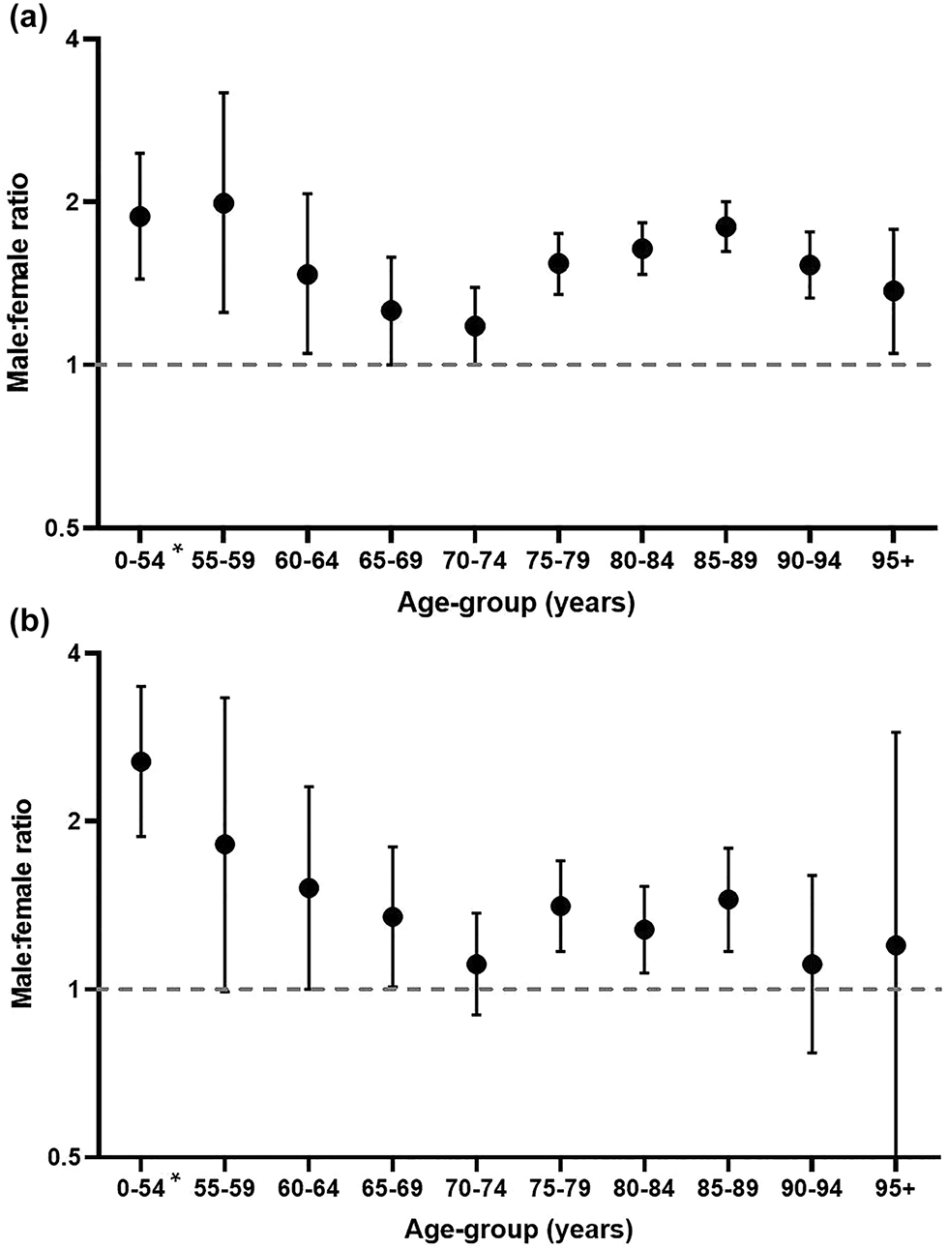
Male to female ratios of case fatality among all notified **a** cases excluding healthcareworkers and among hospitalized **b** cases stratified by age group. Ratio calculated by dividing mortality among notified cases in males by mortality among notified cases in females. *ages between 0 and 55 have been aggregated due to low numbers

Of all cases notified by May 31st, 6070 died (20.5%). Mortality among notified cases was 22.9% for males and 18.3% for females (OR of 1.33 [95% CI 1.26–1.41]). Of hospitalized cases, 2466 died (22.0%), also with a significant difference between males (23.5%) and females (19.4%); OR of 1.27 [95% CI 1.16–1.40]. For both males and females, the case fatality rate among all cases as well as among hospitalised cases increased with age (supplement B). For all age-groups, case fatality was higher among males than among females, for all notified cases and for hospitalized cases (Fig. 1). Odds ratios adjusted for differences in age were higher than the unadjusted odds ratios (Table 2). Supplementing this age-adjusted model with the comorbidities did not change the odds ratios. We found cardiovascular disease or hypertension, diabetes, chronic neuromuscular disease, renal impairment, chronic lung disease and malignancy to be independent risk factors for mortality, also for both sexes separately (supplement C). Adding nursing home residency to the model slightly increased the adjusted odds ratio for case fatality.

**Table 2:** Crude and adjusted odds ratios for case fatality comparing males to females among all notified cases and among hospitalized cases (February 27th–May 31st)

	Cases	Unadjusted		Adjusted (age)		Adjusted (+ comorbidity)		Adjusted (+ nursing home-residency)	
		OR	[95% CI]	OR	[95% CI]	OR	[95% CI]	OR	[95% CI]
All cases									
Total	29,539	1.33	[1.26–1.41]	1.83	[1.72–1.95]	NA		1.91	[1.79–2.03]^2^
Cases with known comorbidity status*	13,344	1.30	[1.20–1.42]	1.65	[1.51–1.80]	1.68	[1.53–1.84]^1^	1.76	[1.60–1.93]^3^
Hospitalizations									
Total	11,227	1.27	[1.16–1.40]	1.45	[1.31–1.61]	NA		1.49	[1.34–1.65]^2^
Cases with known comorbidity status*	7567	1.38	[1.23–1.55]	1.50	[1.33–1.70]	1.54	[1.36–1.74]^1^	1.56	[1.38–1.77]^3^

Model variants: (1) Sex + Age + Comorbidity; (2) Sex + Age + Nursing home residency; (3) Sex + Age + Comorbidity + nursing home residency

^*^Subgroup in which data on comorbidities was available. Data on comorbidities was only reported consistently until April 10th

**Fig. 2:**
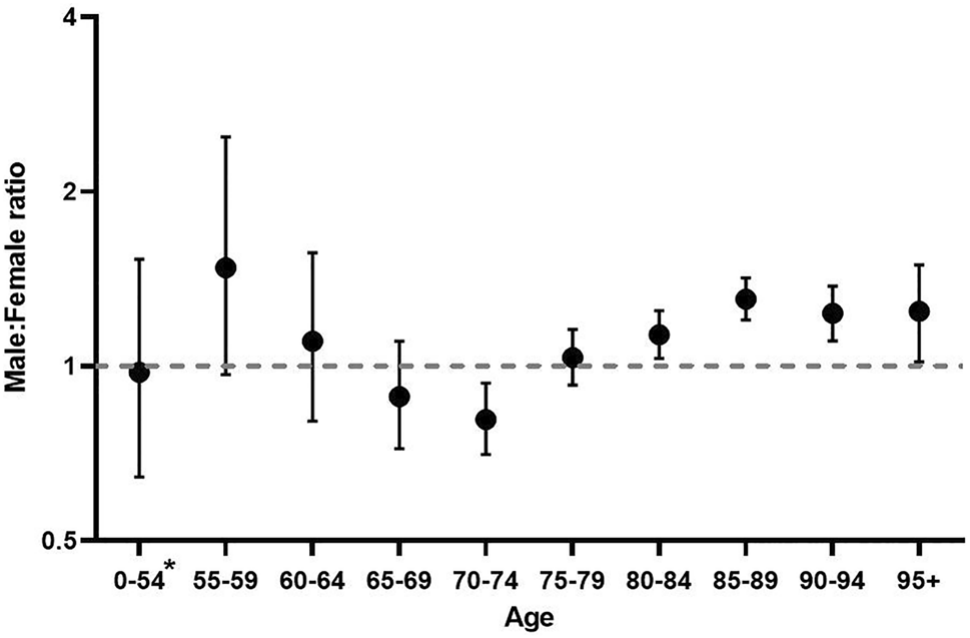
Male to female ratio of age-adjusted standardized mortality ratio (case fatality adjusted for population mortality). *ages between 0 and 55 have been aggregated due to low numbers

The standardized mortality ratio (SMR) among notified COVID-19 cases was 4.8 in males [95%CI 4.64–4.96] and 2.8 in females [95%CI 2.71–2.93] with a male–female ratio of 1.7 [95%CI 1.62–1.78]. Stratified by age, this male–female difference was only significant above one for age-groups above 80 years (Fig. 2). Remarkably, the point estimate of the SMR was highest in the 55–59 year-age group (1.48 [95% CI 0.97–2.48]) but significantly below one among 70–74 years old (SMR 0.81 [95%CI 0.70–0.94]).

### COVID-19 mortality in the population

**Fig. 3:**
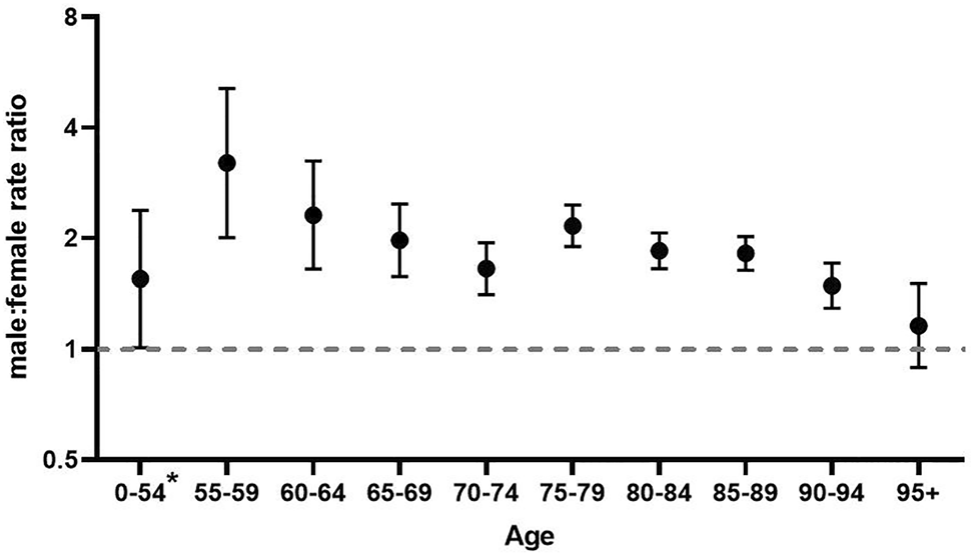
Male–female population mortality rate ratio of COVID-19 cases

**Fig. 4:**
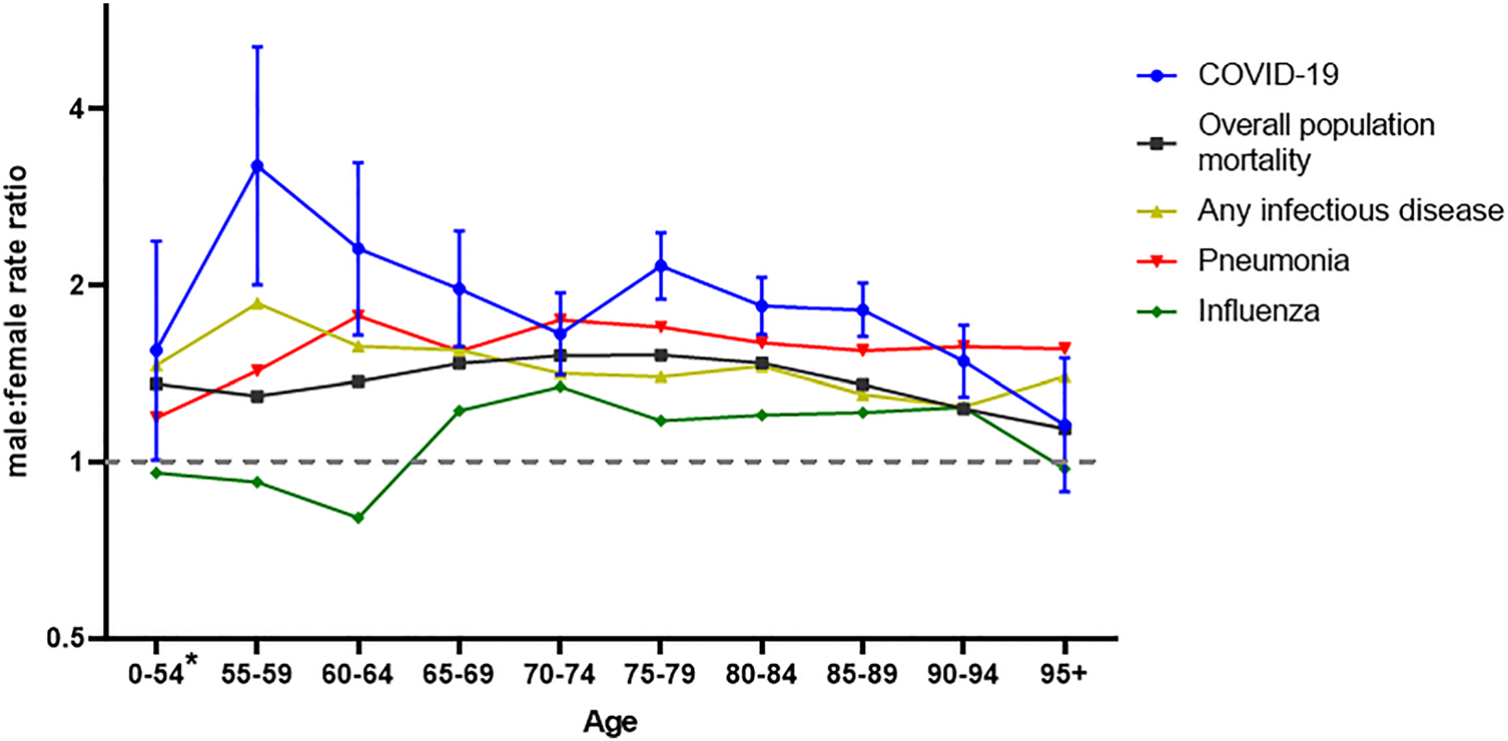
Male–female population mortality rate ratio of COVID-19 cases compared to the overall population mortality rate ratio in the Dutch population, the pneumonia related mortality rate (ICD10: J12-J18), the influenza related mortality rate (J09-J11), and the mortality rate related to infectious diseases in general (ICD10: A00-B99). General population mortality specific mortality is calculated over the past 5 years, 2015–2019 and based on death certificates. Other confidence intervals are shown in supplement F

The peak in total COVID-19 deaths was much higher for males than females and was reached towards the end of March, slightly earlier for males than females. The number of deaths decreased faster in males than in females and after April 8th, the daily number of reported deaths was slightly higher in females (supplement D). The majority of deaths in females consisted of nursing home residents, a group in which the number of deaths increased more slowly. The population mortality rate was 35.1 per 100.000, with a male–female rate ratio of 1.25 [95% CI 1.18–1.31]. Among nursing-home residents only, the male–female mortality rate ratio was 1.88 [95%CI 1.74–2.02]. When stratified by age, the absolute number of deaths was higher in males younger than 90 years than females younger than 90 (supplement E). Number of deaths in males was highest in the age group 75–80 years (739 deaths) and in females in the age group 85–89 years (663 deaths). Mortality rates increased by age in both sexes (supplement F). In all age-groups from 55 to 94 years, the population mortality rates were significantly higher (range 1.53 – 3.21) in males than females. In the age-group 95 + years (with low population numbers) and in the age-group 0–54 years (with few COVID-19 deaths), the rate was also higher in males, but not significantly so (Fig. 3). Between the ages of 55–69 and 75–94 years, male–female ratios for COVID-19 mortality were significantly higher than for overall mortality in the general population. Also compared to mortality due to any infectious disease in general, male–female ratio was higher, though this was only significant between the age of 75 and 89. Male–female discrepancies in COVID-19 mortality were less distinct from pneumonia mortality and only significant in the age-group 55–59 and 75–79 (Fig. 4, supplement F).

## Discussion

We report a higher case fatality in males among COVID-19 notified cases including hospitalized cases which cannot be explained by differences in age-distribution or the prevalence of comorbidities. COVID-19 related mortality increased with increasing age and was higher in males in every age group. Relative to the expected mortality in the general population, as reflected by the SMR, the risk of dying was also higher in male cases, suggesting that mortality differences have a COVID-19 specific component. However, when stratified by age, this COVID-19 specific effect could only be confirmed for cases aged 80 years or older. Strikingly, for the age-group 70–74 years, the standardized mortality ratio was higher in females, probably mainly due to the low mortality relative to males in the general population. In perspective of general population numbers, COVID-19 mortality strongly increased with age for both males and females and was higher in males among all age-groups. Differences in COVID-19 population mortality rates were greatest for the age-group 55–59 years but were lower in the oldest age-groups. Male–female discrepancies in mortality are not a unique phenomenon, as has been reported by Nielsen et al. [22]. We showed that population mortality rates for overall mortality, mortality due to infectious disease and mortality due to pneumonia and influenza are also higher in males. However, male–female ratio was even higher for COVID-19 mortality. The observed male–female difference in mortality can only partially be attributed to sex-differences observed in mortality in general, indicating that additional COVID-19 specific factors contribute to these sex-discrepancies.

Hospitalization was found to be more frequent in males. In addition, the National Intensive Care Evaluation foundation (NICE) reported the vast majority of COVID-19 related ICU-patients to be males (71.8% vs. 28.2%, n = 2876). As well, in accordance with our findings, these ICU reports on COVID-19 indicate a higher risk of mortality among males admitted to the ICU (32.6%) than among females (23.7%) with an odds ratio of 1.66 [95% CI 1.27–1.89] [23]**.**

Although many countries report sex-disaggregated data on COVID-19 mortality, only few countries describe these differences in perspective of their population numbers and population-level mortality rates and in relation to other factors including age and comorbidity [18, 19]. Worldwide, the CFR among confirmed COVID-19 cases is 1.4 times higher in men [12]. Gebhard et al. reported male–female CFR-ratios in several European countries and China between 1.7 and 1.8. In our study we found a 1.3 times higher risk of mortality among male cases during the first 3 months of the outbreak in the Netherlands. However, with the inclusion of healthcare workers this ratio increases to 1.8, illustrating the influence of testing policy and gender-role factors on these nationwide data.

Population mortality rate ratios are less affected by testing policies than the CFR because deaths are related to known population size denominators, which are independent of case notification. However, the number of COVID-19 deaths in the numerator is still underestimated due to incomplete testing and incomplete reporting among notified cases and it is unclear if there is a gender-bias in the tested and/or unreported cases. Furthermore, mortality rates do not purely reflect mortality risk but also include the risk of infection. Age-specific population mortality rates in the Netherlands show a similar pattern as in other countries, where an exponential increase with age with higher mortality rates in males has been reported [24, 25]. Standardized mortality ratios have not yet been reported by other countries. However, sex-discrepancies in population mortality have been described to be higher for COVID-19 than for all-cause mortality, confirming a COVID-19 specific effect on mortality [25].

There are various biological mechanisms described that could contribute to COVID-19 specific sex differences. Immune response differs between males and females in several areas due to, among others, X-chromosomal dependent and hormonal driven mechanisms [14, 26–28]**.** The influence of sex-hormones has also been pointed out as a contributing factor in sex discrepancies observed in mortality by SARS-CoV-1 and MERS [29]**.** More specific for COVID-19, a study comparing differences in SARS-CoV-2 IgG antibodies found that in severe COVID-19 cases relatively higher IgG antibody levels were observed in females indicating a better antibody response against SARS-CoV2 in females [30]**.** Another interesting finding is the dysfunction in the TRL7 gene located on the X-chromosome in young severely affected male COVID-19 patients, resulting in immunological defects in two types of interferons, crucial in viral immune response [31]. Besides immunological processes, the involvement of the ACE-II receptor in cell penetration of SARS-COV-2 has been described as a factor likely contributing to sex discrepancies in COVID-19 severity because of sex differences in expression due to the location of this enzyme on the X-chromosome [14, 32, 33].

Although we excluded healthcare workers, gender-related factors are likely to influence mortality. Health-seeking behavior generally tends to be higher in females, which may lead to earlier initiation of treatment, potentially resulting in a milder disease course [34]. However, the restricted testing policies during the first wave made this less likely to be of significant impact. A systematic review on behavioral changes during and after the 2009 influenza pandemic showed that women were more likely to follow recommended measures such as hygiene regulations [35]**.** These gender-related factors remain difficult to quantify and cannot be fully distinguished from biological factors.

In addition, mortality differences seem to decrease with increasing age. Though not as evident in our data, this was reported by several other studies [14, 18, 24, 25]. Public health England described a twofold higher chance of dying from COVID-19 in males compared to females of working population age (aged between 20 and 65 years), while this risk of death was only 1.5 times higher in older adults (aged above 65) [19]. A study describing excess mortality during March 2020 in Lombardy, Italy (a region severely affected by the COVID-19 pandemic) reported excess mortality —based on national mortality data during the COVID-19 outbreak — to be higher in males. This difference decreased in older age-groups [18]. Though our data also show a decrease in mortality differences with older age, confidence intervals are wide in the younger age-groups due to low mortality. Furthermore, male–female differences are noticeably smaller around the age of 70, with even a higher SMR in females.

The data used for the analysis described here were based on surveillance of mandatory notifications, which provided a large dataset, but with suboptimal accuracy, especially during the peak of the pandemic in the regions most affected. Deaths in COVID-19 patients were registered without uniform criteria on whether death was directly caused by COVID-19. A major limitation is the underreporting of mortality in residential institutions. Due to testing policies in nursing homes, initially often only two–three symptomatic patients were tested per nursing home unit. Given that the majority of nursing home residents are women, this testing policy has likely led to underestimation of COVID-19 population mortality rate in elderly women. However, the effect of sex on mortality among notified cases is possibly underestimated by the underreporting of cases in nursing homes. As already mentioned, testing strategies during the first months of the pandemic undeniably caused selection bias. Excluding healthcare workers from the analysis reduced this selection bias, but excluded a larger part of the female population at risk compared with males because healthcare workers are more likely to be female [21]. This excluded population is expected to have a low risk of mortality since it is relatively healthy and contains only 1795 people with an age above 60 years (7.7% of the total amount of notified cases above 60). When it comes to comorbidities, the multivariable logistic regression did not account for obesity, while this is more prevalent in men and has been indicated as a major risk factor for severe COVID-19 outcomes.

It is evident that sex plays a role in COVID-19 severity, likely influenced by a combination of sex- and gender-dependent factors. Though several hypotheses on contributing biological processes have been proposed, few studies describe sex differences in relation to age and comorbidities.

Our study confirms male sex is a predisposing factor for severe outcomes of COVID-19, independent of age and comorbidities, and that additional to COVID-19 specific mechanisms likely contribute to this mortality discrepancy. More knowledge is required regarding the nature of these underlying mechanisms and how this could inform the prioritization and personalization of treatment for COVID-19 based on sex.

## Supplementary Information

Below is the link to the electronic supplementary material.

Supplementary file1 (DOCX 193 KB)

## Data Availability

Rough characteristics of all positively tested cases have been made publicly available on case-based level [36]. However, due to privacy regulations, the full dataset used for this study cannot be made available.

## References

[1] Coronavirus disease (COVID-19) [Internet]. [cited 2020 Jul 19]. Available from: https://www.who.int/emergencies/diseases/novel-coronavirus-2019?gclid=Cj0KCQjw3s_4BRDPARIsAJsyoLORtdy3VOMLIb2xx7qK5b_hH-Tm9vZBZUYJE_Fm4FnYTtOp-K-WaR0aAtJ1EALw_wcB

[2] Wu C, Chen X, Cai Y, Xia J, Zhou X, Xu S, et al. Risk factors associated with acute respiratory distress syndrome and death in patients with coronavirus disease 2019 pneumonia in Wuhan, China. JAMA Intern Med [Internet]. 2020 [cited 2020 Jul 19]; 180:934. Available from: https://jamanetwork.com/journals/jamainternalmedicine/fullarticle/2763184 10.1001/jamainternmed.2020.0994 PMC7070509 32167524

[3] Docherty AB, Harrison EM, Green CA, Hardwick HE, Pius R, Norman L, et al. Features of 20 133 UK patients in hospital with covid-19 using the ISARIC WHO clinical characterisation protocol: prospective observational cohort study. BMJ [Internet]. 2020 [cited 2020 Jul 19]; 369. Available from: /pmc/articles/PMC7243036/?report=abstract 10.1136/bmj.m1985 PMC7243036 32444460

[4] Petrilli CM, Jones SA, Yang J, Rajagopalan H, O’Donnell LF, Chernyak Y, et al. Factors associated with hospitalization and critical illness among 4103 patients with COVID-19 disease in New York City. medRxiv [Internet]. 2020 [cited 2020 Jul 19]; 2020.04.08.20057794. Available from: 10.1101/2020.04.08.20057794

[5] Onder G, Rezza G, Brusaferro S. Case-fatality rate and characteristics of patients dying in relation to COVID-19 in Italy [Internet]. Vol. 323, JAMA - Journal of the American Medical Association. American Medical Association; 2020 [cited 2020 Jul 19]. p. 1775–6. Available from: https://www.who.int/docs/default- 10.1001/jama.2020.4683 32203977

[6] Flodgren GM, Vestrheim DF, Brurberg KG. COVID-19: risk factors associated with disease severity – a rapid review, 2nd update. [Covid-19 og risikofaktorer for alvorlig sykdom - en hurtigoversikt, andre oppdatering. Hurtigoversikt 2020] Oslo: Norwegian Inst 2020. M e mo. Heal (San Fr. 2011; 9637–9637)

[7] Petrilli CM, Jones SA, Yang J, Rajagopalan H, O’Donnell L, Chernyak Y, et al. Factors associated with hospital admission and critical illness among 5279 people with coronavirus disease 2019 in New York City: prospective cohort study. BMJ [Internet]. 2020; 369. Available from: https://www.bmj.com/content/369/bmj.m1966 10.1136/bmj.m1966 PMC7243801 32444366

[8] Guan W, Ni Z, Hu Y, Liang W, Ou C, He J (2020). Clinical characteristics of coronavirus disease 2019 in China. N Engl J Med.

[9] Chen N, Zhou M, Dong X, Qu J, Gong F, Han Y (2020). Epidemiological and clinical characteristics of 99 cases of 2019 novel coronavirus pneumonia in Wuhan, China: a descriptive study. Lancet [Internet].

[10] Karlberg J, Chong DSY, Lai WYY. Do men have a higher case fatality rate of severe acute respiratory syndrome than women do? Am J Epidemiol Hopkins Bloom Sch Public Heal All rights Reserv [Internet]. 2004 [cited 2020 Jul 20]; 159(3):229–31. Available from: http://www.info.gov.hk/info/sars/e_news.htm 10.1093/aje/kwh056 PMC7110237 14742282

[11] Chen X, Chughtai AA, Dyda A, MacIntyre CR (2017). Comparative epidemiology of Middle East respiratory syndrome coronavirus (MERS-CoV) in Saudi Arabia and South Korea. Emerg Microbes Infect [Internet].

[12] Global Health 50/50 [Internet]. 2020. 2020 [cited 2020 Feb 8]. Available from: https://globalhealth5050.org/the-sex-gender-and-covid-19-project/

[13] Van Oyen H, Nusselder W, Jagger C, Kolip P, Cambois E, Robine JM. Gender differences in healthy life years within the EU: an exploration of the “health-survival” paradox. Int J Public Health [Internet]. 2013 [cited 2020 Jul 20]; 58:143–55. Available from: /pmc/articles/PMC3557379/?report=abstract 10.1007/s00038-012-0361-1 PMC3557379 22618297

[14] Gebhard C, Regitz-Zagrosek V, Neuhauser HK, Morgan R, Klein SL. Impact of sex and gender on COVID-19 outcomes in Europe [Internet]. Vol. 11, Biology of sex differences. BioMed Central Ltd.; 2020 [cited 2020 Jul 19]. Available from: https://pubmed.ncbi.nlm.nih.gov/32450906/10.1186/s13293-020-00304-9 PMC7247289 32450906

[15] Bhopal R. Covid-19 worldwide: we need precise data by age group and sex urgently [Internet]. Vol. 369, The BMJ. BMJ Publishing Group; 2020 [cited 2020 Jul 20]. Available from: http://group.bmj.com/group/rights-licensing/10.1136/bmj.m1366 32245830

[16] P C, A Y. Coronavirus COV-19/SARS-CoV-2 affects women less than men: clinical response to viral infection. J Biol Regul Homeost Agents [Internet]. 2020 [cited 2020 Jul 20]; 34. Available from: https://pubmed.ncbi.nlm.nih.gov/32253888/10.23812/Editorial-Conti-3 32253888

[17] Jin J-M, Bai P, He W, Wu F, Liu X-F, Han D-M (2020). Gender differences in patients with COVID-19: focus on severity and mortality. Front Public Heal [Internet].

[18] Marcon G, Tettamanti M, Capacci G, Fontanel G, Spanò M, Nobili A (2020). COVID-19 mortality in Lombardy: the vulnerability of the oldest old and the resilience of male centenarians. Aging (Albany NY).

[19] Public Health England. Disparities in the risk and outcomes of COVID-19, PHE publications gateway number: GW-1447, August 2020. Available from: https://assets.publishing.service.gov.uk/government/uploads/system/uploads/attachment_data/file/908434/Disparities_in_the_risk_and_outcomes_of_COVID_August_2020_update.pdf

[20] LCI, RIVM. LCI richtlijn COVID-19. 2020/05/14 [Internet]. Available from: https://lci.rivm.nl/richtlijnen/covid-19

[21] Statistics Netherlands (CBS), 2020 [Internet]. Available from: https://opendata.cbs.nl/statline/#/CBS/en/

[22] Nielsen J, Nørgaard SK, Lanzieri G, Vestergaard LS, Moelbak K (2021). Sex-differences in COVID-19 associated excess mortality is not exceptional for the COVID-19 pandemic. Sci Rep [Internet].

[23] COVID-19 in Dutch Intensive Care Units; Patient characteristics and outcomes compared with pneumonia patients in the ICU from 2017–2019. Version 2020-07-14.

[24] Spiegelhalter D (2020). Use of “normal” risk to improve understanding of dangers of covid-19. BMJ.

[25] Ng J, Bakrania K, Russell R, Falkous C. COVID-19 mortality rates by age and gender: why is the disease killing more men than women? Rgare [Internet]. 2020; 215(July):1–14. Available from: https://www.rgare.com/knowledge-center/media/research/covid-19-mortality-rates-by-age-and-gender-why-is-the-disease-killing-more-men-than-women

[26] Gemmati D, Bramanti B, Serino ML, Secchiero P, Zauli G, Tisato V (2020). COVID-19 and individual genetic susceptibility/receptivity: role of ACE1/ACE2 genes, immunity, inflammation and coagulation. Might the double X-chromosome in females be protective against SARS-COV-2 compared to the single X-chromosome in males?. Int J Mol Sci.

[27] Klein SL, Flanagan KL. Sex differences in immune responses [Internet]. Vol. 16, Nature reviews immunology. Nature Publishing Group; 2016 [cited 2020 Jul 20]. p. 626–38. Available from: www.nature.com/nri 10.1038/nri.2016.90 27546235

[28] Scully EP, Haverfield J, Ursin RL, Tannenbaum C, Klein SL (2020). Considering how biological sex impacts immune responses and COVID-19 outcomes. Nat Rev Immunol [Internet].

[29] Channappanavar R, Fett C, Mack M, Ten Eyck PP, Meyerholz DK, Perlman S. Sex-based differences in susceptibility to severe acute respiratory syndrome coronavirus infection. J Immunol [Internet]. 2017 [cited 2020 Jul 19]; 198: 4046–53. Available from: /pmc/articles/PMC5450662/?report=abstract 10.4049/jimmunol.1601896 PMC5450662 28373583

[30] Zeng F, Dai C, Cai P, Wang J, Xu L, Li J, et al. A comparison study of SARS-CoV-2 IgG antibody between male and female COVID-19 patients: a possible reason underlying different outcome between sex. J Med Virol [Internet]. 2020 [cited 2020 Jul 20]; Available from: /pmc/articles/PMC7267228/?report=abstract 10.1002/jmv.25989 PMC7267228 32383183

[31] Van Der Made CI, Simons A, Schuurs-Hoeijmakers J, Van Den Heuvel G, Mantere T, Kersten S (2020). Presence of genetic variants among young men with severe COVID-19. JAMA–J Am Med Assoc.

[32] Gheblawi M, Wang K, Viveiros A, Nguyen Q, Zhong JC, Turner AJ, et al. Angiotensin-converting enzyme 2: SARS-CoV-2 receptor and regulator of the renin-angiotensin system: celebrating the 20th anniversary of the discovery of ACE2 [Internet]. Vol. 126, Circulation research. Lippincott Williams and Wilkins; 2020 [cited 2020 Jul 20]. p. 1456–74. Available from: www.ahajournals.org/journal/res 10.1161/CIRCRESAHA.120.317015 PMC7188049 32264791

[33] Patel SK, Velkoska E, Burrell LM (2013). Emerging markers in cardiovascular disease: where does angiotensin-converting enzyme 2 fit in?. Clin Exp Pharmacol Physiol [Internet].

[34] Thompson AE, Anisimowicz Y, Miedema B, Hogg W, Wodchis WP, Aubrey-Bassler K (2016). The influence of gender and other patient characteristics on health care-seeking behaviour: a QUALICOPC study. BMC Fam Pract [Internet]..

[35] Tooher R, Collins JE, Street JM, Braunack-Mayer A, Marshall H. Community knowledge, behaviours and attitudes about the 2009 H1N1 influenza pandemic: a systematic review. Influenza Other Respi Viruses [Internet]. 2013 [cited 2020 Jul 20]; 7:1316–27. Available from: /pmc/articles/PMC4634241/?report=abstract https://data.rivm.nl/geonetwork/srv/dut/catalog.search#/metadata/2c4357c8-76e4-4662-9574-1deb8a73f724?tab=relations 10.1111/irv.12103 PMC4634241 23560537

