# Supplementary material for: Sex differences in COVID-19 mortality in the Netherlands
Source: Infection. 2022 Feb 9;50(3):709–17. doi: 10.1007/s15010-021-01744-0 (PMC9151564; doi:10.1007/s15010-021-01744-0)

# Supplementary material

**A. Handling of missing values**


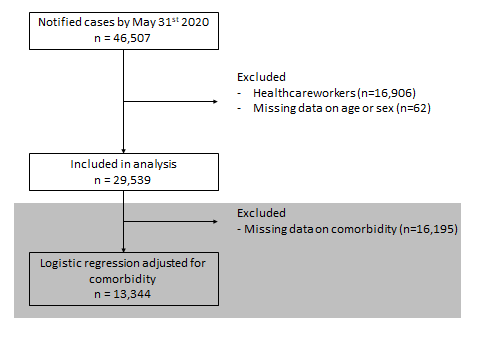


**B. Case fatality rates by age-group for all notified COVID-19 cases (excluding health care workers) N=29,539, and for hospitalized COVID-19 cases, N=11,227.**


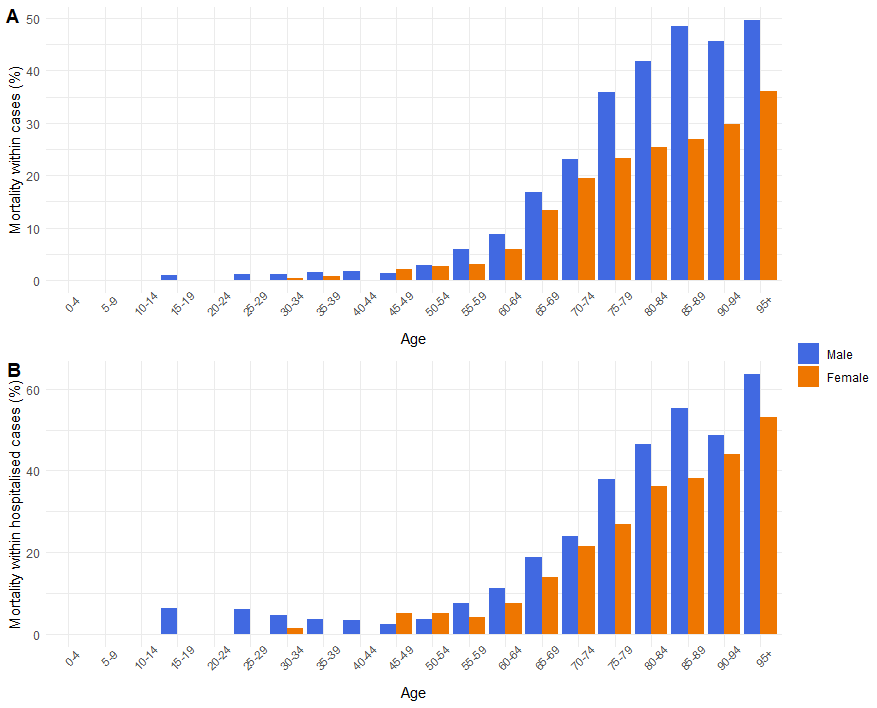


**C. Comorbidities as risk factor for death among notified male and female cases, adjusted for differences in age**

|  | **Males** | | **Females** | | **Total** |  |
| --- | --- | --- | --- | --- | --- | --- |
|  | *OR* | *95% CI* | *OR* | *95% CI* | *OR* | *95%CI* |
| **Cardiovascular disease or hypertension** | 1.14 | 0.99-1.31 | 1.20 | 1.07-1.35 | 1.17 | 1.07-1.28 |
| **Diabetes** | 1.42 | 1.20-1.67 | 1.36 | 1.17-1.57 | 1.38 | 1.24-1.54 |
| **Liver disease** | 1.00 | 0.43-2.11 | 1.19 | 0.63-2.15 | 1.08 | 0.66-1.73 |
| **Chronic neuromuscular disease** | 1.55 | 1.27-1.87 | 2.25 | 1.87-2.71 | 1.87 | 1.64-2.13 |
| **Immunodeficiency** | 0.77 | 0.32-1.63 | 0.91 | 0.49-1.61 | 0.85 | 0.52-1.34 |
| **Renal impairment** | 1.48 | 1.18-1.86 | 1.53 | 1.24-1.88 | 1.51 | 1.30-1.76 |
| **Chronic lung disease** | 1.30 | 1.10-1.53 | 1.18 | 1.02-1.36 | 1.23 | 1.10-1.37 |
| **Malignancy** | 1.50 | 1.18-1.89 | 1.38 | 1.15-1.67 | 1.44 | 1.24-1.66 |
| **Other underlying disease** | 1.15 | 0.96-1.36 | 1.27 | 1.08-1.48 | 1.22 | 1.08-1.37 |

**D. Reported COVID-19 deaths per day, moving average over 14 days**


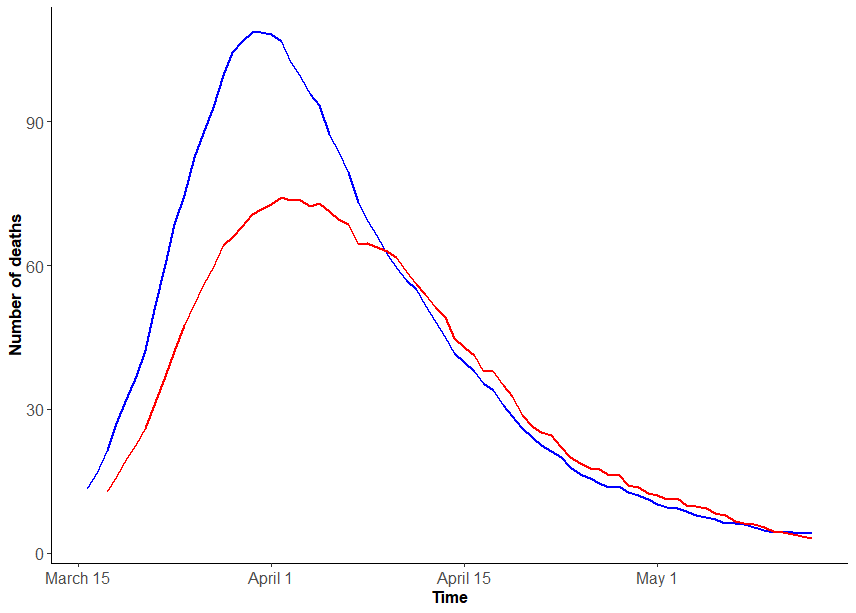


|  | Males |
| --- | --- |
|  | Females |

**Reported COVID-19 deaths per day stratified by household**

**
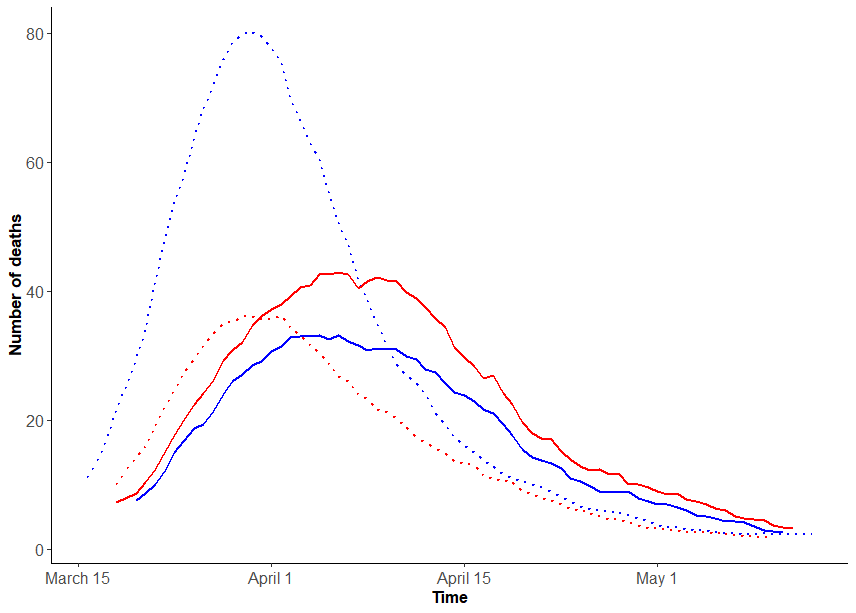
**

**E. Absolute number of deaths in notified COVID-19 cases (figure A) and population mortality rates per 100.000 inhabitants (figure B)**


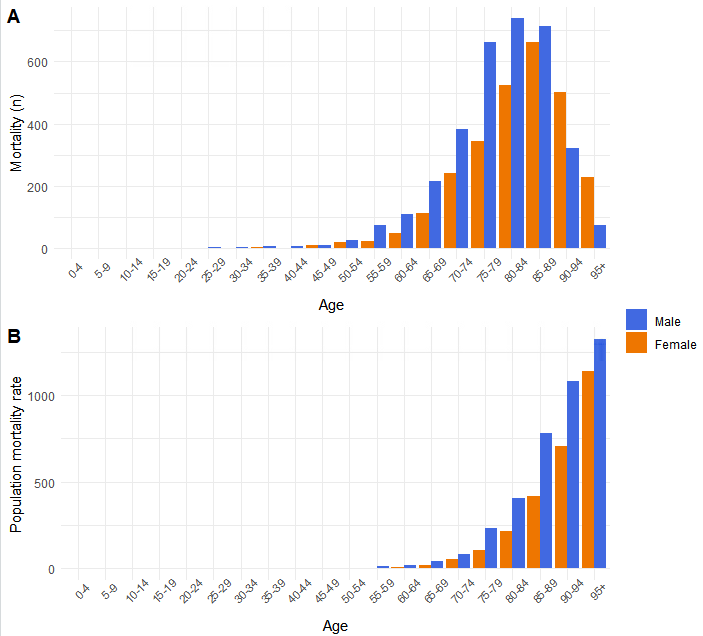


*A) Absolute number of deaths by age, B) Mortality rate per 100.000 Dutch inhabitants, by age*

**F. Male-female mortality rate ratios compared to mortality rate ratios of different causes of death**


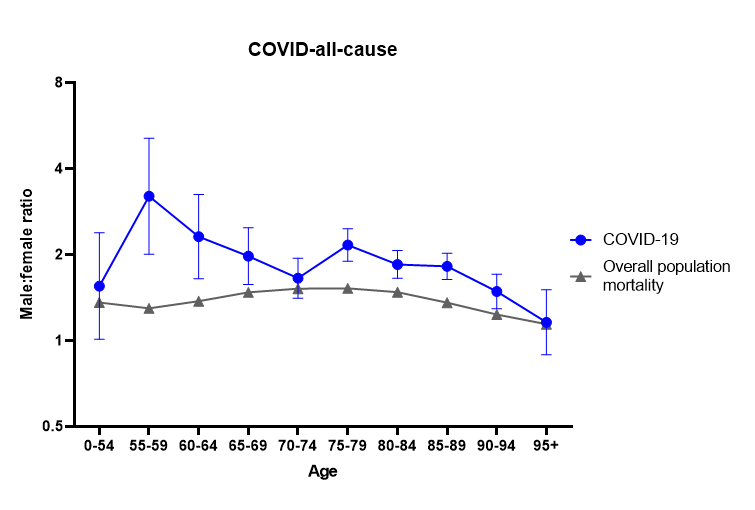

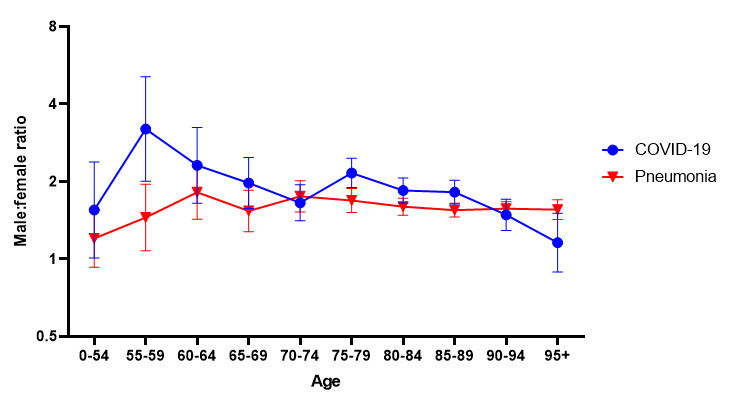

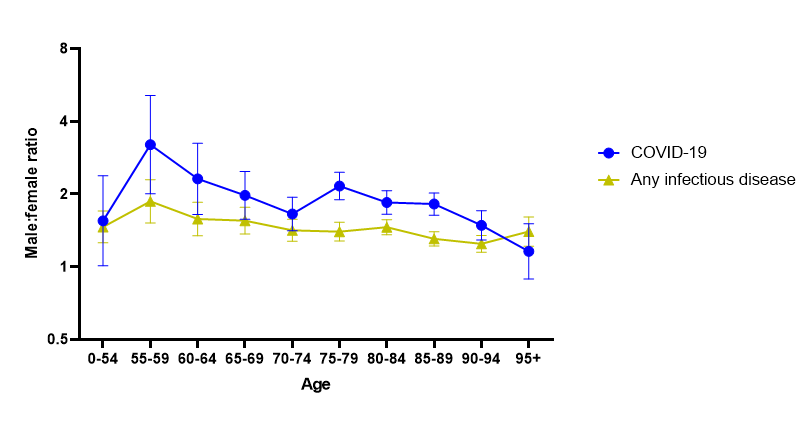


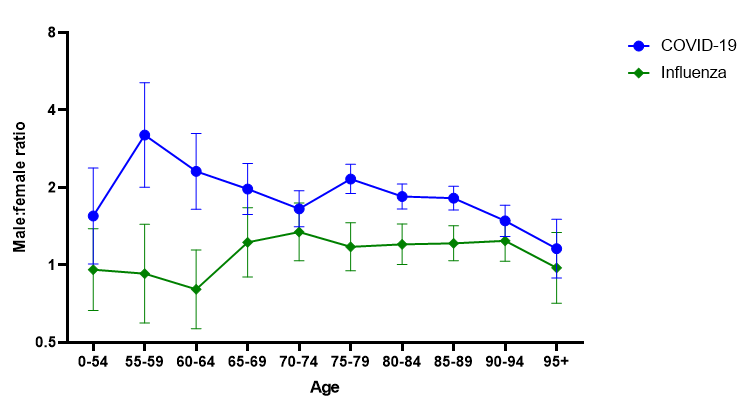

Supplement: Supplementary file 1 — Supplementary file1 (DOCX 193 KB) [file 15010_2021_1744_MOESM1_ESM.docx]
